# A new nomogram for predicting extraurothelial recurrence in patients with upper urinary tract urothelial carcinoma following radical nephroureterectomy

**DOI:** 10.3389/fonc.2024.1442168

**Published:** 2024-11-06

**Authors:** Hao Wu, Dan Jia, Xianyu Dai, Hongliang Cao, Fulin Wang, Tong Yang, Lei Wang, Tao Xu, Baoshan Gao

**Affiliations:** Department of Urology II, The First Hospital of Jilin University, Changchun, China

**Keywords:** upper urinary tract urothelial carcinoma, radical nephroureterectomy, extra-urinary recurrence, prediction model, online network calculator

## Abstract

**Purpose:**

We sought to develop and validate a nomogram for predicting extra-urinary recurrence (EUR) following radical nephroureterectomy (RNU) in patients with upper urinary tract urothelial carcinoma (UTUC).

**Methods:**

Data from 556 UTUC patients post-RNU at the First Hospital of Jilin University were retrospectively analyzed. These patients were categorized into a training group (n=389) and a validation group (n=167). Variables significantly associated with prognosis were identified using univariate Cox regression and most minor absolute shrinkage and selection operator (LASSO) analysis. These independent predictors were incorporated into the nomogram to estimate extra-urinary recurrence-free survival (EURFS). Validation of the nomogram involved ROC curves, calibration plots, and decision curve analysis (DCA). Patients were stratified into two risk categories based on their nomogram scores to compare EURFS using the Kaplan-Meier method.

**Results:**

Eight predictors were identified: T-stage, N-stage, tumor grade, local and nerve invasion, preoperative hemoglobin level, neutrophil-to-lymphocyte ratio (NLR), and creatinine, all proving to be independent predictors of EUR. A nomogram was created based on these eight factors, and using the ROC, calibration curves, and DCA, good prediction results were shown in both the training and validation groups. The training and validation groups also showed reliable predictive performance. In particular, there was a significant difference in survival between the high-risk and low-risk groups (P<0.0001). We have also built a network calculator that calculates patient survival time. The URL is https://haowu24.shinyapps.io/dynnomapp.

**Conclusion:**

A nomogram for predicting distant metastases in UTUC patients was successfully developed, and its accuracy, reliability, and clinical value were demonstrated. This new tool helps to improve the clinical management of UTUC cases.

## Introduction

Upper urinary tract uroepithelial carcinoma (UTUC) is a relatively uncommon malignancy, comprising 7-8% of all urological tumors and 5% of all uroepithelial malignancies (1). Siegel et al. (2) observed a gender disparity in UTUC incidence, with a 2:1 male-to-female ratio. The established gold standard for UTUC management is radical nephroureterectomy, which includes nephroureterectomy combined with a cystectomy sleeve (3). Although RNU is the recommended treatment, high recurrence and metastasis rates remain a significant concern, especially in the advanced stages of UTUC.

UTUC is often detected at advanced stages and is associated with a dismal prognosis (4). Studies have demonstrated that even following radical nephroureterectomy, the rates of recurrence and metastasis for UTUC range between 22% and 66% (5, 6). Recurrences may occur locally, distally, in the bladder, or contralaterally, reflecting the aggressive nature of these tumors (7). The 5-year intravesical recurrence-free survival rate (IVRFS) is estimated to be between 46% and 54% (8). Moreover, postoperative complications include extravesical recurrences, which occur in about 27.3-33% of cases and involve lymph nodes, distal organs, and the original tumor site. These are referred to as extra-urinary recurrences (EUR) (9), and their 5-year survival rate for high-risk patients is notably lower at 36.2% (9). This highlights the aggressive nature of EUR and its distinct management challenges compared to intravesical recurrences.

The prognosis for UTUC patients is contingent upon factors across the preoperative, intraoperative, and postoperative stages. While most research focuses on intravesical recurrence risk following radical nephroureterectomy, factors such as gender, prior bladder cancer, chronic kidney disease, positive preoperative urine cytology, ureteral tumor location, tumor multifocality, and aggressive pathological staging (infiltrative pT) have been identified (10, 11). However, despite their clinical significance, there is a lack of research focusing on EUR-specific risk factors. Early identification of patients at high risk for EUR could greatly benefit treatment planning and monitoring.

Patients experiencing EUR tend to have poorer outcomes. Despite their importance, studies on EUR remain sparse (12), underscoring the need for enhanced predictive models to guide clinical decisions, including selecting more aggressive treatments and appropriate monitoring strategies (13). Unlike intravesical recurrence, EUR poses unique challenges in management and prognosis, further emphasizing the need for specialized risk stratification tools. In response, we conducted a retrospective analysis of demographic, pathological, and clinical data from 700 patients at the First Hospital of Jilin University. From this data, we developed and validated a nomogram model for EUR to identify and promptly address risk factors, improving patient management and outcomes. This nomogram aims to provide clinicians with a practical and reliable tool to predict EUR, helping tailor treatment strategies for high-risk patients.

## Methods

### Patient selection and follow-up

**Figure 1:**
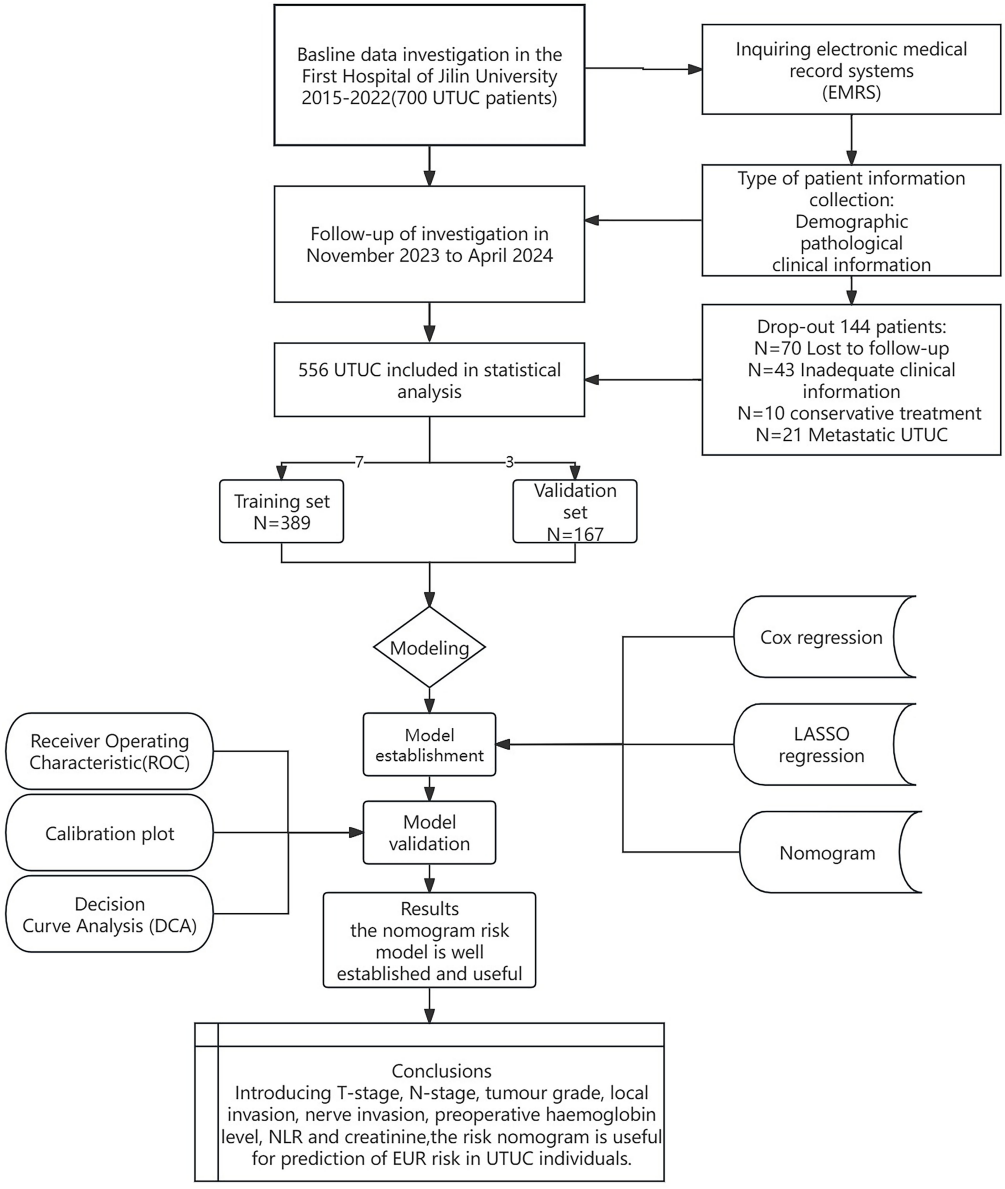
Flow diagram of study design.

We conducted a retrospective analysis of UTUC patients who underwent RNU from January 2015 to December 2022 at the First Hospital of Jilin University, China. The Institutional Research Ethics Committee approved this study, which adhered to the STROCSS guidelines (14). All participants provided informed consent. All procedures conformed to relevant regulations and the Declaration of Helsinki. Trained clinicians performed all diagnostic and assessment activities. **Figure 1** shows a flow diagram of the study design.

Inclusion criteria were as follows: (1) patients diagnosed with primary and pathologically confirmed UTUC; (2) patients with comprehensive clinical records; (3) patients with unilateral disease; (4) patients who underwent RNU with a cystectomy sleeve. Exclusion criteria included: (1) patients with bilateral UTUC; (2) patients who did not receive RNU with a cystectomy sleeve; (3) patients with metastatic uroepithelial carcinoma; (4) patients lacking complete clinical data; (5) patients with congenital urological anomalies. None of the enrolled patients received neoadjuvant chemotherapy.

Patient data from the First Hospital of Jilin University were randomly assigned to training and validation cohorts in a 7:3 ratio. Follow-up occurred every three months for the first two years post-surgery and subsequently every six months up to 5 years. Evaluations included comprehensive histories, clinical examinations, serum and urine tests, physical examinations, urinary ultrasound, chest and abdominal CT scans, cystoscopic evaluations, and urine cytology. Additional imaging tests, such as magnetic resonance imaging (MRI), bone scans, or positron emission tomography-computed tomography (PET-CT), were performed based on clinical indications. This study did not categorize intravesical and contralateral upper urinary tract recurrences as extraendothelial recurrences. Overall survival (OS) was defined as the period from RNU to death from any cause. Extra-urothelial recurrence-free survival (EURFS) was measured from the surgery date to the occurrence of EUR. The neutrophil-to-lymphocyte ratio (NLR), lymphocyte-to-monocyte ratio (LMR), and platelet-to-lymphocyte ratio (PLR) were also assessed.

### Data collection

Demographic and clinical variables, such as age, gender, body mass index (BMI), history of hypertension, diabetes mellitus, bladder cancer, tumor side, pathological stage (pT), lymph node status (pN0, pNx, or pN+), tumor grade, size, multifocality, margin status, lymphovascular invasion (LVI), urocytology, hydrocele, and hematological parameters including NLR, PLR, and LMR, were recorded or calculated from clinical records at the time of RNU.

In this study, to ensure the robustness and reliability of the model, variance inflation factor (VIF) was used to detect multicollinearity among the variables (**Supplementary Table 1**). All VIF values were within an acceptable range (below 3), indicating that there was no significant multicollinearity between the variables. This ensures that high correlations between variables do not compromise the model’s explanatory power and predictive accuracy.

All surgical specimens underwent standard pathological processing, and a genitourinary pathologist reviewed each section. Tumor staging adhered to the American Joint Committee on Cancer (AJCC) TNM classification (8th edition), and grading followed the 2016 World Health Organization (WHO) criteria. Tumor location referred to the primary tumor site. The presence of tumors or abnormal cells in preoperative urine samples was documented as positive urine cytology.

### Statistical analysis

Continuous variables with normal distribution are reported as mean ± standard deviation, while those not normally distributed are presented as medians with interquartile ranges. Categorical variables are expressed as n (%). Statistical significance was assessed using two-tailed tests, with p values < 0.05 deemed significant. Variable selection was based on one-way COX regression analysis, most minor absolute shrinkage, and selection operator (LASSO) regularisation. To minimize overfitting, ten-fold cross-validation was utilized. LASSO coefficients for selected variables were nonzero at λmin, indicating potential independent risk factors. Independent risk factors were incorporated into graphs. EURFS at 1, 3, and 5 years was displayed using column-line plots. The model’s discriminative ability was evaluated with a consistency index (C-index), and calibration curves assessed model calibration.

Decision curve analysis (DCA) was calculated to evaluate the clinical utility of the nomogram model compared to predictions based solely on pathological tumor staging, assessing the accuracy of both the nomogram model and pathological T-staging. Patients’ EURFS was predicted using a nomogram, and a total risk score was calculated for each patient in the training and validation cohorts to classify them into high and low-risk groups. Differences in survival curves between these groups were analyzed in the training cohort and validated using Kaplan-Meier (K-M) analysis in the validation cohort. All assessment methods were applied in both the training and validation sets. P-values were two-tailed, and p < 0.05 was considered statistically significant. All statistical analyses and graphs were conducted using R software (version 4.2.2) and IBM SPSS Statistics (version 24).

## Results

### Patient characteristics

Following the inclusion and exclusion criteria, 556 patients with pathologically confirmed UTUC were included. Of these, 189 (34.0%) experienced EUR. Patients were categorized into a no-recurrence group (n=367) and a recurrence group (n=189) based on the occurrence of EUR. The average age of all participants was 68.9 years, with 58.8% being female. Recurrence was associated with older age, lower hemoglobin levels, shorter follow-up periods, a higher choice of surgical approach for NLR, advanced pathological T-stages, regional lymph node status, tumor grade, infiltration degree, neurological invasion, and elevated creatinine levels in the recurrent group compared to the non-recurrent group, with a statistically significant difference (p < 0.05) (**Table 1**).

**Table 1:** Clinical and pathological features of patients with or without EUR.

	[ALL] N=556	No-EUR N=367	EUR N=189	P value
Sex:				0.629
Female	327 (58.8%)	219 (59.7%)	108 (57.1%)	
Male	229 (41.2%)	148 (40.3%)	81 (42.9%)	
Age	68.9 (10.1)	67.8 (9.74)	70.9 (10.5)	0.001
Hematuria:				0.584
No	161 (29.0%)	103 (28.1%)	58 (30.7%)	
Yes	395 (71.0%)	264 (71.9%)	131 (69.3%)	
Surgical Procedure:				0.015
Abdominal	85 (15.3%)	59 (16.1%)	26 (13.8%)	
Retroperitoneal	415 (74.6%)	262 (71.4%)	153 (81.0%)	
Robot_assisted	56 (10.1%)	46 (12.5%)	10 (5.29%)	
Concurrent Bladder Tumor at Diagnosis:				0.740
No	519 (93.3%)	344 (93.7%)	175 (92.6%)	
Yes	37 (6.65%)	23 (6.27%)	14 (7.41%)	
Multifocality:				1.000
No	479 (86.2%)	316 (86.1%)	163 (86.2%)	
Yes	77 (13.8%)	51 (13.9%)	26 (13.8%)	
Size	3.57 (2.28)	3.47 (2.20)	3.77 (2.42)	0.157
T:				0.001
≤2	356 (64.0%)	253 (68.9%)	103 (54.5%)	
≥3	200 (36.0%)	114 (31.1%)	86 (45.5%)	
N:				0.017
0	523 (94.1%)	352 (95.9%)	171 (90.5%)	
≥1	33 (5.94%)	15 (4.09%)	18 (9.52%)	
Side:				0.318
Left	309 (55.6%)	210 (57.2%)	99 (52.4%)	
Right	247 (44.4%)	157 (42.8%)	90 (47.6%)	
Location:				0.627
Renal pelvis	253 (45.5%)	164 (44.7%)	89 (47.1%)	
Ureteral	234 (42.1%)	154 (42.0%)	80 (42.3%)	
Renal pelvic- Ureteral junction	69 (12.4%)	49 (13.4%)	20 (10.6%)	
Grade:				0.001
Low-grade	52 (9.35%)	46 (12.5%)	6 (3.17%)	
High-grade	504 (90.6%)	321 (87.5%)	183 (96.8%)	
Invasion:				0.001
No	43 (7.73%)	39 (10.6%)	4 (2.12%)	
Yes	513 (92.3%)	328 (89.4%)	185 (97.9%)	
NI:				0.006
No	497 (89.4%)	338 (92.1%)	159 (84.1%)	
Yes	59 (10.6%)	29 (7.90%)	30 (15.9%)	
HB	123 (19.3)	125 (19.1)	120 (19.4)	0.006
NLR	2.96 (2.86)	2.61 (2.30)	3.64 (3.63)	<0.001
PLR	149 (99.4)	148 (102)	153 (94.4)	0.573
LMR	4.57 (4.38)	4.49 (3.58)	4.74 (5.63)	0.575
Creatinine	119 (125)	106 (103)	145 (158)	0.003
Blood Type:				0.495
A	139 (25.0%)	90 (24.5%)	49 (25.9%)	
AB	49 (8.81%)	37 (10.1%)	12 (6.35%)	
B	198 (35.6%)	127 (34.6%)	71 (37.6%)	
O	170 (30.6%)	113 (30.8%)	57 (30.2%)	
Surgical Duration	162 (57.3)	164 (57.7)	156 (56.3)	0.116
EURT	45.6 (24.3)	52.4 (23.4)	32.3 (20.3)	<0.001

NI, nerve invasion; HB, hemoglobin; PLR, platelet-to-lymphocyte ratio; NLR, neutrophil-to-lymphocyte ratio; LMR, lymphocyte-to-monocyte ratio; EURT, EUR follow-up time.

The patients were divided into a training set (n=389) and a validation set (n=167) in a 7:3 ratio. The mean follow-up duration was 43.9 months for the training set and 49.5 months for the validation set. Most variables were similar between the training and validation sets, except for the later onset of EUR in the validation set compared to the training set (49.5 months vs. 43.9 months, p<0.05). There was no statistical difference in the duration of follow-up between the two groups. The clinicopathological characteristics of the patients are detailed in **Table 2**.

**Table 2:** Clinical and pathological characteristics of patients.

	[ALL] (N=556)	Training set (N=389)	Validation set (N=167)	P value
Sex:				0.238
Female	327 (58.8%)	222 (57.1%)	105 (62.9%)	
Male	229 (41.2%)	167 (42.9%)	62 (37.1%)	
Age	68.9 (10.1)	68.9 (10.1)	68.8 (10.3)	0.920
Hematuria:				0.431
No	161 (29.0%)	117 (30.1%)	44 (26.3%)	
Yes	395 (71.0%)	272 (69.9%)	123 (73.7%)	
Surgical Procedure:				0.664
Abdominal	85 (15.3%)	60 (15.4%)	25 (15.0%)	
Retroperitoneal	415 (74.6%)	287 (73.8%)	128 (76.6%)	
Robot_assisted	56 (10.1%)	42 (10.8%)	14 (8.38%)	
Concurrent Bladder Tumor at Diagnosis:				1.000
No	519 (93.3%)	363 (93.3%)	156 (93.4%)	
Yes	37 (6.65%)	26 (6.68%)	11 (6.59%)	
Multifocality:				0.713
No	479 (86.2%)	337 (86.6%)	142 (85.0%)	
Yes	77 (13.8%)	52 (13.4%)	25 (15.0%)	
Size	3.57 (2.28)	3.55 (2.22)	3.63 (2.40)	0.692
T:				0.934
≤2	356 (64.0%)	250 (64.3%)	106 (63.5%)	
≥3	200 (36.0%)	139 (35.7%)	61 (36.5%)	
N:				0.534
0	523 (94.1%)	368 (94.6%)	155 (92.8%)	
≥1	33 (5.94%)	21 (5.40%)	12 (7.19%)	
Side:				0.538
Left	309 (55.6%)	220 (56.6%)	89 (53.3%)	
Right	247 (44.4%)	169 (43.4%)	78 (46.7%)	
Location:				0.397
Renal pelvis	253 (45.5%)	176 (45.2%)	77 (46.1%)	
Ureteral	234 (42.1%)	169 (43.4%)	65 (38.9%)	
Renal pelvic-ureteral junction	69 (12.4%)	44 (11.3%)	25 (15.0%)	
Grade:				0.191
Low-grade	52 (9.35%)	41 (10.5%)	11 (6.59%)	
High-grade	504 (90.6%)	348 (89.5%)	156 (93.4%)	
Invasion:				1.000
No	43 (7.73%)	30 (7.71%)	13 (7.78%)	
Yes	513 (92.3%)	359 (92.3%)	154 (92.2%)	
NI:				0.714
No	497 (89.4%)	346 (88.9%)	151 (90.4%)	
Yes	59 (10.6%)	43 (11.1%)	16 (9.58%)	
HB	123 (19.3)	123 (19.5)	125 (19.0)	0.269
NLR	2.96 (2.86)	3.01 (2.62)	2.85 (3.36)	0.594
PLR	149 (99.4)	151 (103)	146 (90.9)	0.530
LMR	4.57 (4.38)	4.59 (4.99)	4.53 (2.43)	0.845
Creatinine	119 (125)	114 (110)	130 (154)	0.229
Blood Type:				0.861
A	139 (25.0%)	100 (25.7%)	39 (23.4%)	
AB	49 (8.81%)	34 (8.74%)	15 (8.98%)	
B	198 (35.6%)	140 (36.0%)	58 (34.7%)	
O	170 (30.6%)	115 (29.6%)	55 (32.9%)	
Surgical Duration	162 (57.3)	162 (56.8)	160 (58.5)	0.711
EUR:				0.304
No	367 (66.0%)	251 (64.5%)	116 (69.5%)	
Yes	189 (34.0%)	138 (35.5%)	51 (30.5%)	
EURT	45.6 (24.3)	43.9 (24.4)	49.5 (23.8)	0.012

NI, nerve invasion; HB, hemoglobin; PLR, platelet-to-lymphocyte ratio; NLR, neutrophil-to-lymphocyte ratio; LMR, lymphocyte-to-monocyte ratio; EUR, extraurothelial recurrence; EURT, EUR follow-up time.

### Variable screening and construction of nomogram

**Figure 2:**
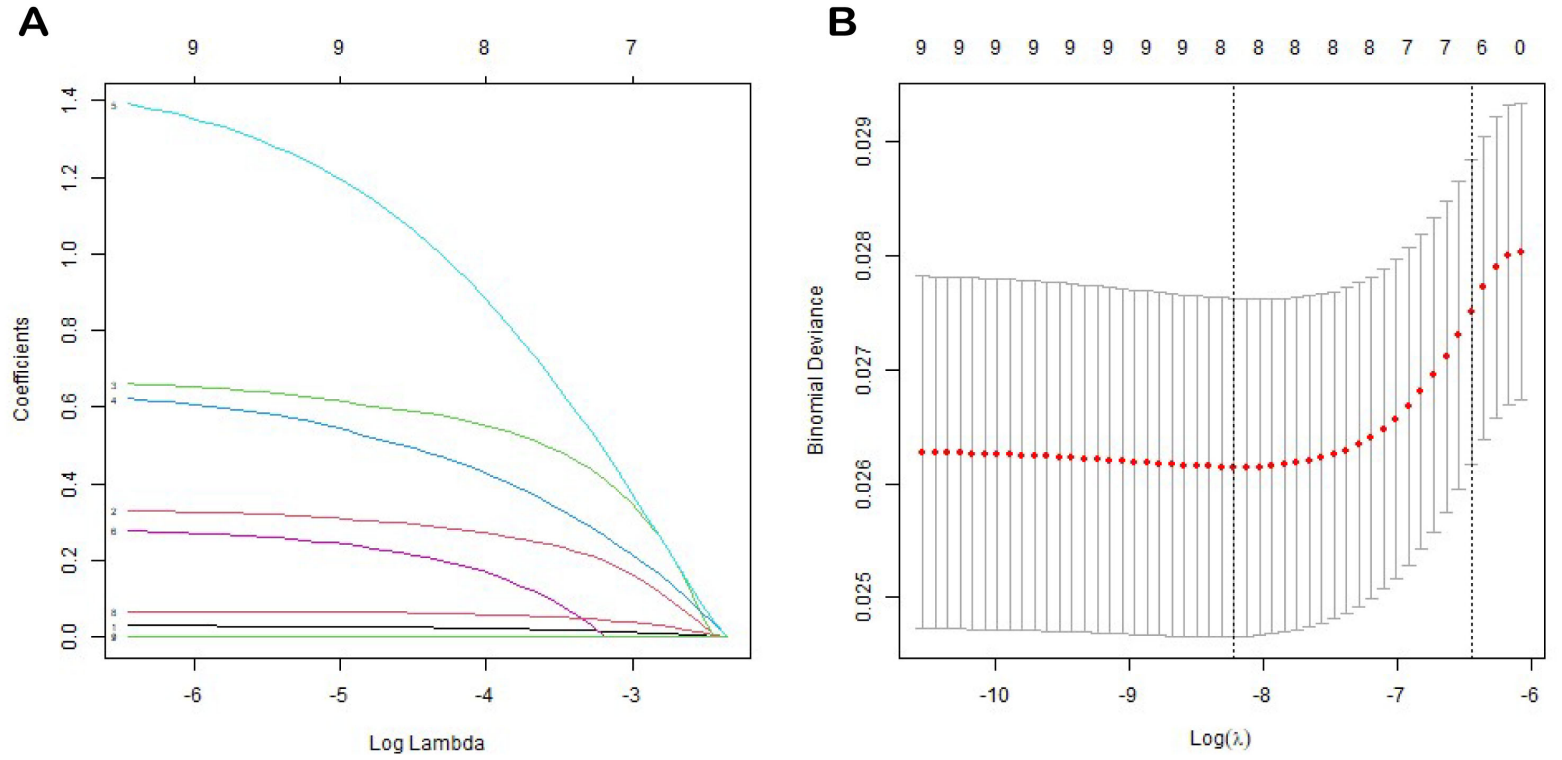
LASSO coefficient profiles of all variables predicting EURFS **(A)**. Tenfold cross-validation for tuning parameter selection in the least LASSO model related to EURFS **(B)**.

We employed a one-way Cox regression with the LASSO model to identify significant predictors of EUR from the training cohort, identifying eight potential predictors with nonzero coefficients (**Figure 2**), as presented in **Table 3**: T stage, N stage, tumor grade, local invasion, neurological invasion, preoperative hemoglobin level, NLR, and creatinine. These factors were established as independent predictors of EUR.

**Table 3:** Results of COX regression analysis.

Variate	uHR(95%CI)	P value
Sex		
Female	ref	
Male	1.1 (0.75-1.5)	0.775
Age	1 (1-1)	0.001
Hematuria		
No	ref	
Yes	0.87 (0.61-1.2)	0.434
Surgical Procedure		
Abdominal	ref	
Retroperitoneal	0.82 (0.5-1.4)	0.43
Robot_assisted	0.87 (0.39-1.9)	0.731
Concurrent Bladder Tumor at Diagnosis		
No	ref	
Yes	1.3 (0.66-2.4)	0.481
Multifocality		
No	ref	
Yes	1.1 (0.68-1.8)	0.694
Size	1.1 (0.98-1.1)	0.16
T		
≤2	ref	
≥3	1.7 (1.2-2.3)	0.003
N		
0	ref	
≥1	2.6 (1.4-4.5)	<0.001
Side		
Left	ref	
Right	1.1 (0.77-1.5)	0.645
Location		
Renal pelvis	ref	
Ureteral	0.89 (0.63-1.3)	0.507
Renal pelvic- ureteral junction	0.7 (0.39-1.3)	0.251
Grade		
Low-grade	ref	
High-grade	3.1 (1.4-7)	0.005
Invasion		
No	ref	
Yes	6.2 (1.5-25)	0.008
NI		
No	ref	
Yes	1.6 (0.97-2.5)	0.096
HB	0.99 (0.98-1)	0.04
NLR	1.1 (1-1.1)	0.004
PLR	1 (1-1)	0.897
LMR	1 (0.97-1)	0.86
Creatinine	1 (1-1)	0.012
Blood Type		
A	ref	
AB	0.59 (0.29-1.2)	0.13
B	1 (0.66-1.5)	0.99
O	0.88 (0.56-1.4)	0.582
Surgical Duration	1 (1-1)	0.343

NI, nerve invasion; HB, hemoglobin; PLR, platelet-to-lymphocyte ratio; NLR, neutrophil-to-lymphocyte ratio; LMR, lymphocyte-to-monocyte ratio.

**Figure 3:**
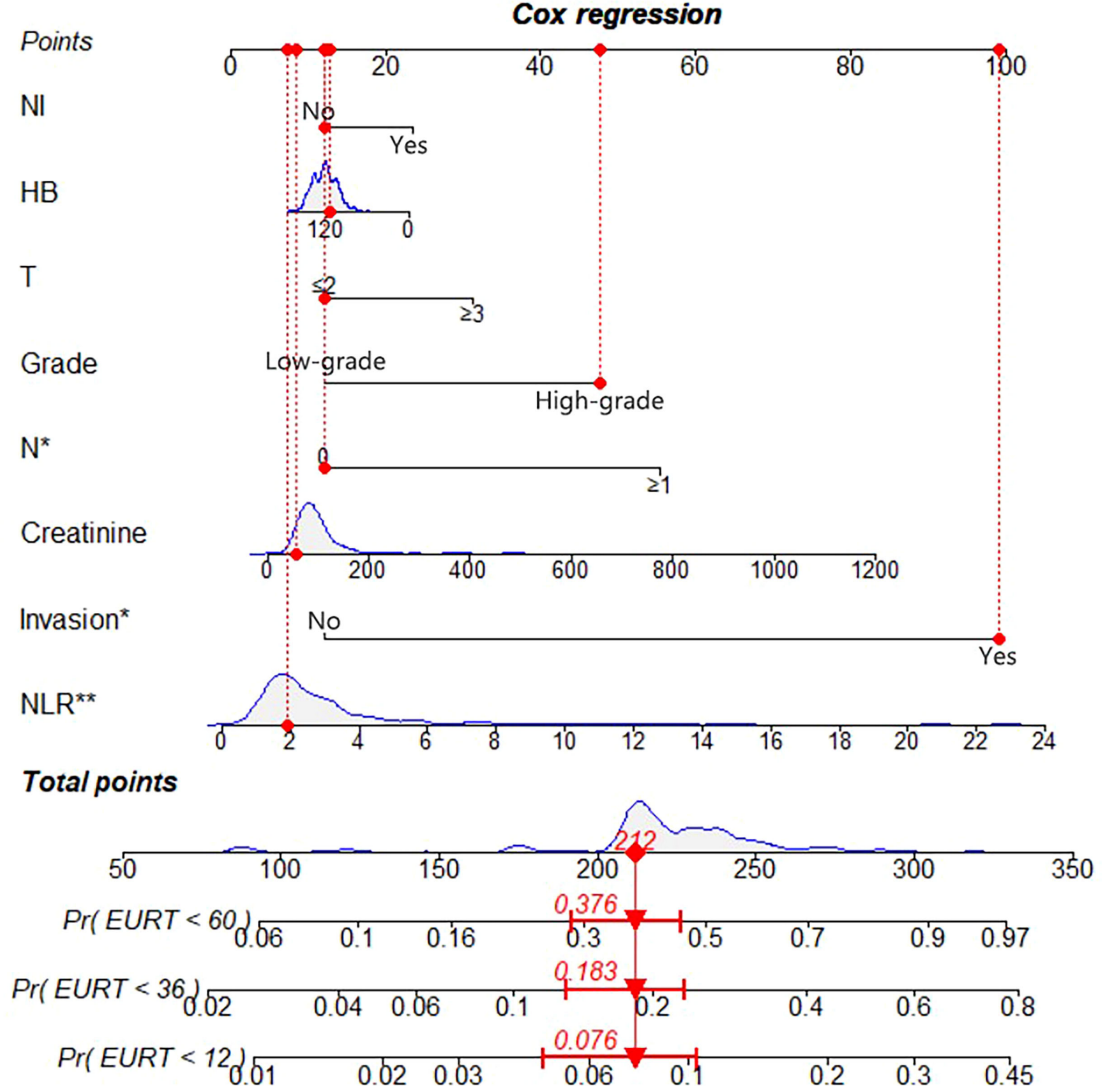
Nomogram for 1-, 3-, and 5-year EURFS prediction of patients with UTUC after RNU.

**Figure 4:**
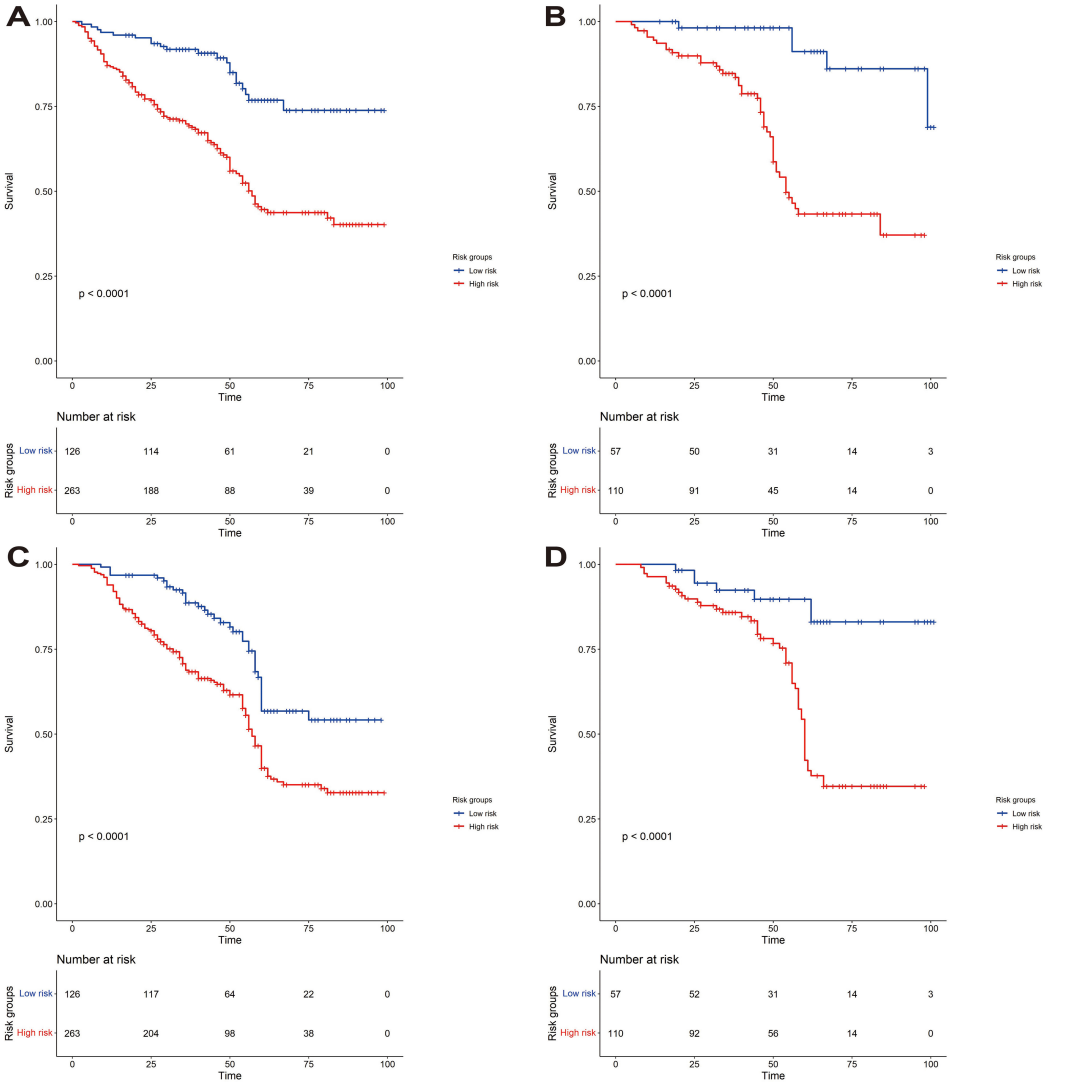
Kaplan-Meier curves for event-free survival analysis for EUR and OS. **(A, B)** Correspond to the Kaplan-Meier curves for the EUR training and validation sets. **(C, D)** are the Kaplan-Meier curves for the OS training and validation sets.

We constructed a nomogram to predict EURFS using these variables, illustrated in **Figure 3**. The total risk score was calculated for each patient in the training and validation cohorts. Patients were classified into high-risk and low-risk groups using the optimal risk score cut-off of 149 (**Figure 4**). K-M survival analysis revealed significantly higher EUR in the high-risk group (P for log-rank < 0.0001).

### Nomogram model performance and validation

**Figure 5:**
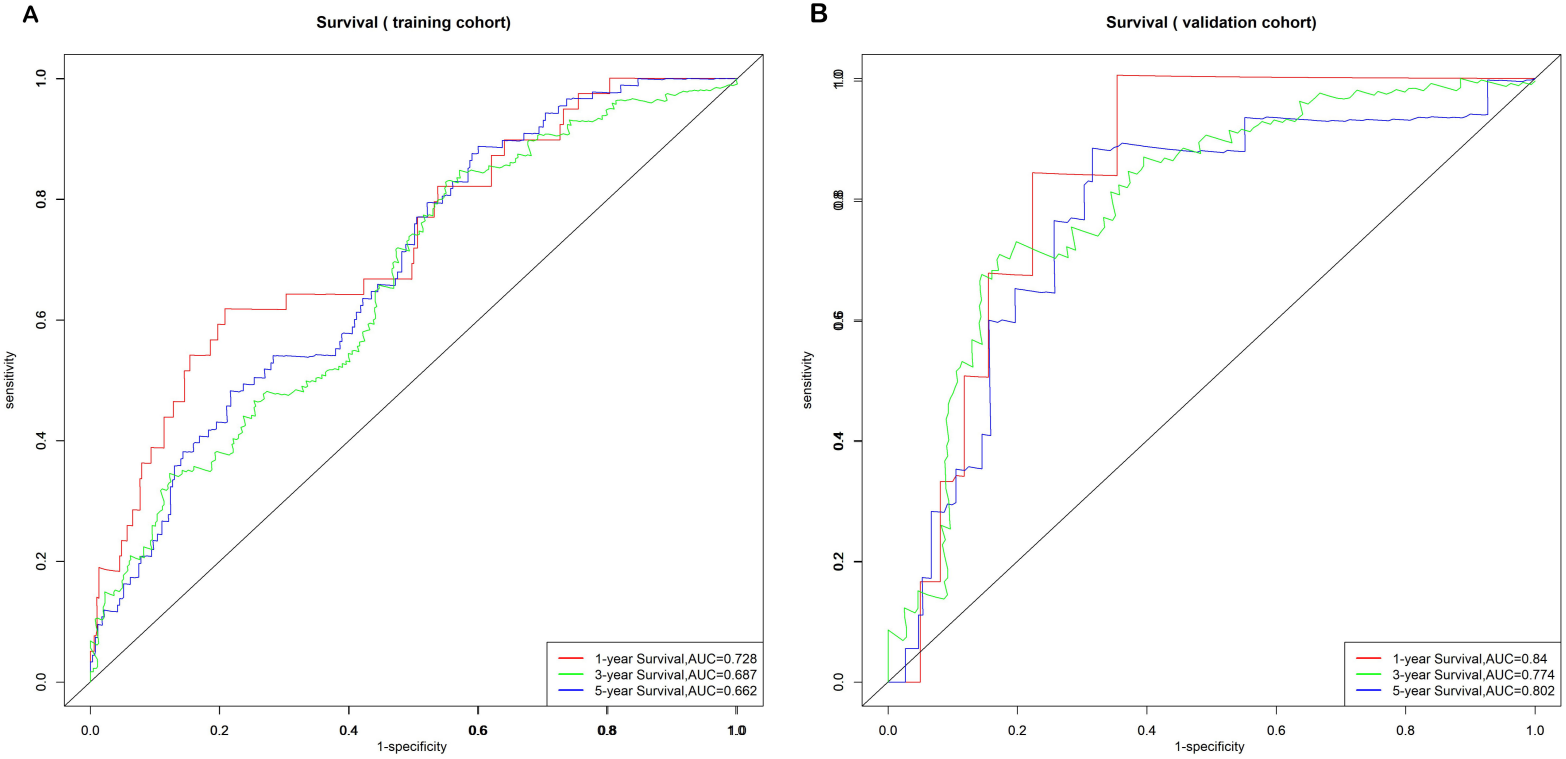
Nomogram ROC curves to predict 1-, 3-, and 5-year EUR in the training cohort **(A)** and validation cohort **(B)**.

**Figure 6:**
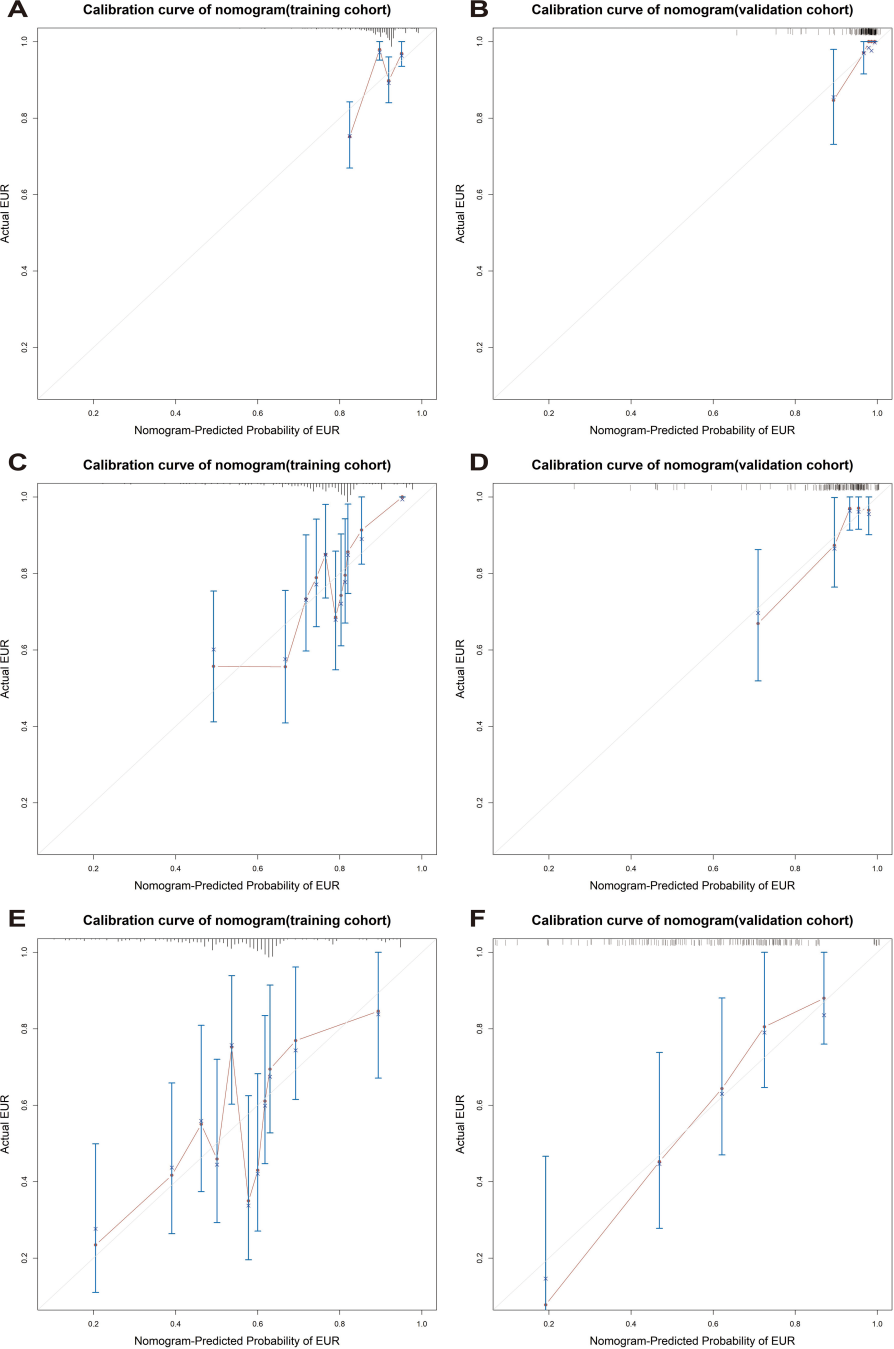
The calibration curves of the EUR nomogram at 1, 3, and 5 years in the training cohort **(A, C, E)** and the validation cohort **(B, D, F)**.

**Figure 7:**
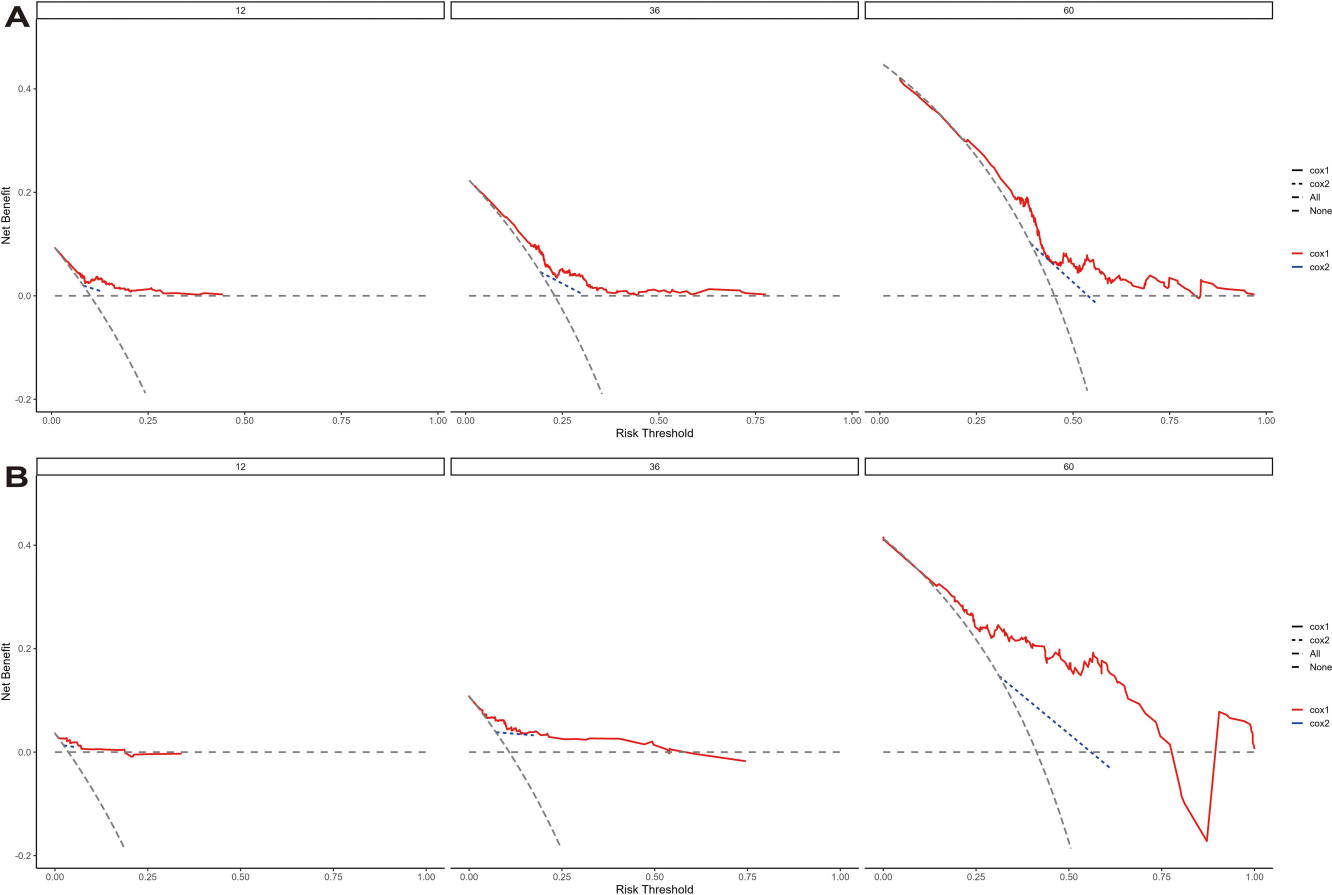
The decision curve analysis of EUR nomogram at 1, 3, and 5 years in the training cohort **(A)** and validation cohort **(B)**. The y-axis measures the net benefit. The thick solid line represents the assumption that all patients have no EUR, and the thin solid line represents the assumption that all patients have EUR. The dotted line represents the risk nomogram.

The model’s discriminative ability was evaluated using the c-index and calibration plots. The c-index was 0.661 for the training set and 0.757 for the validation set. Prediction performance in the training set yielded AUC values of 0.728, 0.687, and 0.662 for 1, 3, and 5 years respectively. In the validation set, AUC values were 0.84, 0.774, and 0.802 for the respective time points (**Figure 5**). Calibration plots for 1, 3, and 5 years demonstrated a good fit (**Figure 6**). Additionally, DCA analysis assessed the model’s predictive performance over these timeframes (**Figure 7**), showing high net benefit in both the training and validation sets, underscoring its predictive efficacy and clinical utility.

### Clinical use of the nomogram

DCA curves further highlighted the superior clinical utility of the nomogram over categorical T-staging in both the training and validation cohorts (**Figure 7**). We calculated risk scores for individuals in both cohorts using the nomogram model. Patients were divided into two groups based on threshold values derived from the training cohort’s risk points. K-M survival curve analysis was conducted to explore the correlation between clinical outcomes and risk group classifications.

### Network calculator

To further improve the applicability of the nomogram model in clinical practice, we developed an online interactive calculator for predicting the risk of extraurinary tract recurrence (EUR) in patients with urothelial carcinoma (UTUC) of the upper tract after radical nephropeterectomy (RNU). The calculator allows users to enter the patient’s vital clinicopathological data, including T stage, N stage, tumor grade, preoperative hemoglobin level, neutrophil-to-lymphocyte ratio (NLR), and other variables, to calculate and display the probability of a patient developing EUR in the next 1, 3 and 5 years.

Using this network calculator, clinicians can quickly assess a patient’s risk of recurrence, leading to a more personalized follow-up plan and treatment strategy for high-risk patients. Compared to traditional manual risk calculations, this online tool is more intuitive and faster, helping to improve the efficiency of clinical decision-making.

The link to the web calculator is https://haowu24.shinyapps.io/dynnomapp.

## Discussion

UTUC is a relatively rare malignancy, representing 5% of all uroepithelial malignancies. It is often diagnosed late and generally has a poor prognosis (15). UTUC is increasingly recognized as a distinct entity, necessitating enhanced efforts from medical stakeholders to understand patient outcomes and their determinants better. Developing predictive models is crucial for patient selection and stratification in clinical trials, providing a valuable tool for physicians when discussing prognosis with patients. While the clinical outcomes of UTUC have been extensively studied, most research has concentrated on bladder recurrences (16); however, the 5-year survival rate for EUR mortality is lower than that for intravesical recurrence-free survival (IVRFS) (9), and studies on EUR are less common. In this study, utilizing numerous clinical samples from the First Hospital of Jilin University, we developed a nomogram to predict the prognosis of UTUC patients undergoing RNU.

T-stage, N-stage, and pathological grading are crucial determinants of postoperative EUR in UTUC. The T-stage is a critical indicator of tumor infiltration depth. Research indicates that patients with stages T3 and T4 face a significantly increased risk of postoperative recurrence, as higher T-stages are generally associated with deeper tumor infiltration and poorer outcomes (17, 18). Lymph node status (N staging) is another vital prognostic factor; the presence of lymph node metastases (N+) typically forecasts poorer postoperative survival and significantly impacts the risk of local and distant recurrences (17, 19). Moreover, the pathological grade of the tumor (e.g., G1, G2, G3) influences recurrence risk; high-grade tumors (e.g., G3) exhibit higher recurrence rates and worse prognosis compared to low-grade tumors (e.g., G1, G2) (18, 20).

UTUC is typically not diagnosed until a patient exhibits visual or microscopic hematuria, the most common symptom of this condition (21). Although it remains unclear whether macroscopic hematuria at diagnosis predicts a worse prognosis than microscopic hematuria, some studies provide partial insights (22). For instance, a retrospective analysis of 532 patients with pathologically confirmed UTUC revealed an approximately 80% increased risk of invasive disease (≥pT2) in patients presenting with gross hematuria (23); additionally, the study by Jakus et al. (24) identified a significant correlation between hematochezia and high-grade or stage ≥T2 UTUC. However, another study involving 179 patients with non-metastatic UTUC found no significant differences in OS or cancer-specific survival (CSS) between patients with or without gross hematuria (25). These findings are consistent with our research, which does not yet confirm that visible hematuria plays a critical role in predicting the prognosis for UTUC patients.

Nerve invasion (NI), characterized by the proliferation of tumor cells along nerve membranes or fibers, indicates a tumor’s invasiveness. A study on a cohort of 803 Chinese patients with non-metastatic UTUC undergoing RNU identified localized neuroinvasion as an independent predictor of poorer progression-free survival (PFS), CSS, and OS (26). This finding aligns with our results, suggesting that patients with NI may require more aggressive postoperative adjuvant therapy and closer follow-up.

Neutrophil/lymphocyte ratio (NLR), platelet/lymphocyte ratio (PLR), and monocyte/lymphocyte ratio (MLR) are widely utilized metrics that reflect systemic inflammation in patients. A meta-analysis encompassing 32 studies determined that an elevated pretreatment NLR independently predicted worse OS (HR=1.72, 95% CI=1.45-2.05), progression-free survival (PFS) (HR=1.68, 95% CI=1.44-1.96), and cancer-specific survival (CSS) (HR=1.64, 95% CI=1.39-1.93) (27). Another meta-analysis corroborated that increased preoperative NLR, PLR, and MLR were significantly linked with adverse outcomes in OS, CSS, disease-free survival/recurrence-free survival/metastasis-free survival (DFS/RFS/MFS), and PFS (28). These findings align with our results, highlighting the pivotal role of inflammation in cancer development and progression (29). Tumors exploit various inflammatory mediators to foster survival, proliferation, and favorable conditions for growth and metastasis (30), making these inflammatory markers valuable for prognostic evaluation.

Hemoglobin and creatinine levels are vital indicators of a patient’s nutritional status and renal function and are crucial for the prognostic assessment of UTUC patients. Low hemoglobin levels may indicate anemia and malnutrition, and preoperative anemia is strongly associated with decreased cancer-specific survival and extra-renal recurrence in UTUC patients post-surgery (13, 31, 32). Adequate renal function is crucial for patients undergoing nephrectomy for UTUC, as it affects postoperative quality of life and long-term survival. Elevated creatinine levels often suggest impaired renal function and a reduced glomerular filtration rate, typical in chronic kidney disease. Morizane et al. (32) found that higher preoperative serum creatinine levels were significantly linked with poorer postoperative CSS in UTUC patients.

Moreover, patients with chronic kidney disease experience higher rates of urothelial cancer-specific mortality (33), and chronic kidney disease is a risk factor for UTUC (34). The aggressiveness of UTUC increases with the severity of CKD (35). However, a meta-analysis involving 657 patients indicated that creatinine levels were not strongly correlated with CSS in UTUC patients (36). This suggests further investigation into the relationship between creatinine and postoperative survival in UTUC patients.

We developed an 8-parameter-based EUR prediction model demonstrating good discriminative ability across the training and external validation sets. The C-index for both predictive models exceeded 0.65, and in the time-dependent ROC analysis, the AUCs at 1, 3, and 5 years were also above 0.65. Furthermore, the calibration curves confirmed that the models were well-calibrated, indicating better net benefit and clinical applicability, as shown on the DCA curve. Patients were stratified into low and high-risk groups according to the nomogram total score, with the K-M method revealing a significant difference in EURFS between revealing groups. In conclusion, our models hold potential value in clinical settings and may assist in assessing the prognosis of UTUC patients following RNU.

While this study offers valuable insights into the under-studied population of patients with distant metastatic UTUC, it has limitations. As a single-center prospective study, its limited diversity may affect the external applicability of the prediction model. Additionally, hospital data constraints prevented the inclusion of genetic or molecular biomarkers. Despite a large sample size, the non-randomized, retrospective design lacks external validation, highlighting the need for more diverse, prospective studies. Future multicenter studies will enhance sample diversity and incorporate new clinical metrics to improve model performance. Despite these limitations, our nomogram performed well in both training and validation cohorts, confirming the reliability of our results.

## Conclusion

We have developed a novel nomogram to predict the prognosis of UTUC patients post-RNU. This tool enables physicians to identify patients at high risk for EUR, thus providing robust support for clinical decision-making and fostering a more personalized treatment approach, ultimately enhancing treatment outcomes.

## Data Availability

The raw data supporting the conclusions of this article will be made available by the authors, without undue reservation.

## References

[1] Ryoo H Kim J Kim T Kang M Jeon HG Jeong BC. Effects of complete bladder cuff removal on oncological outcomes following radical nephroureterectomy for upper tract urothelial carcinoma. Cancer Res Treat. (2021) 53:795–802. doi: 10.4143/crt.2020.919 33421984 PMC8291174

[2] Mousavian A-H Shafiee G Sheidaei A Balajam NZ Ebrahimi M Khatami F. The 15-year national trends of urinary cancers incidence among Iranian men and women; 2005–2020. Int J Equity Health. (2024) 23:13. doi: 10.1186/s12939-023-02084-1 38254127 PMC10804628

[3] Wang C-S Li C-C Juan Y-S Wu W-J Lee H-Y. 5α-reductase inhibitors impact prognosis of urothelial carcinoma. BMC Cancer. (2020) 20. doi: 10.1186/s12885-020-07373-4 PMC7488389 32917158

[4] Blumendeller C Boehme J Frick M Schulze M Rinckleb A Kyzirakos C. Use of plasma ctDNA as a potential biomarker for longitudinal monitoring of a patient with metastatic high-risk upper tract urothelial carcinoma receiving pembrolizumab and personalized neoepitope-derived multipeptide vaccinations: a case report. J ImmunoTherapy Cancer. (2021) 9:e001406. doi: 10.1136/jitc-2020-001406 PMC7802705 33431630

[5] Cheng S Zhong W Xia K Hong P Lin R Wang B. Prognostic role of stromal tumor-infiltrating lymphocytes in locally advanced upper tract urothelial carcinoma: A retrospective multicenter study (TSU-02 study). OncoImmunology. (2021) 10:1861737. doi: 10.1080/2162402X.2020.1861737 33489471 PMC7801121

[6] Margulis V Youssef RF Karakiewicz PI Lotan Y Wood CG Zigeuner R. Preoperative multivariable prognostic model for prediction of nonorgan confined urothelial carcinoma of the upper urinary tract. J Urol. (2010) 184:453–8. doi: 10.1016/j.juro.2010.03.142 20620397

[7] Pan S-Y Huang C-P Chen W-C. Synchronous/metachronous multiple primary Malignancies: review of associated risk factors. Diagnostics. (2022) 12:1940. doi: 10.3390/diagnostics12081940 36010291 PMC9406460

[8] Cutress ML Stewart GD Zakikhani P Phipps S Thomas BG Tolley DA. Ureteroscopic and percutaneous management of upper tract urothelial carcinoma (UTUC): systematic review. BJU Int. (2012) 110:614–28. doi: 10.1111/j.1464-410X.2012.11068.x 22471401

[9] Luo Z Jiao B Yan Y Liu Y Chen H Guan Y. A novel nomogram for predicting extraurothelial recurrence in patients with upper urinary tract urothelial carcinoma after radical nephroureterectomy. J Cancer Res Clin Oncol. (2023) 149:14241–53. doi: 10.1007/s00432-023-05237-5 PMC11796878 37555950

[10] Seisen T Granger B Colin P Léon P Utard G Renard-Penna R. A systematic review and meta-analysis of clinicopathologic factors linked to intravesical recurrence after radical nephroureterectomy to treat upper tract urothelial carcinoma. Eur Urol. (2015) 67:1122–33. doi: 10.1016/j.eururo.2014.11.035 25488681

[11] Lin M-Y Li W-M Huang C-N Lee H-L Niu S-W Chen L-T. Dialysis increases the risk of bladder recurrence in patients with upper tract urothelial cancer: A population-based study. Ann Surg Oncol. (2018) 25:1086–93. doi: 10.1245/s10434-017-6295-3 29330720

[12] Margulis V Shariat SF Matin SF Kamat AM Zigeuner R Kikuchi E. Outcomes of radical nephroureterectomy: a series from the Upper Tract Urothelial Carcinoma Collaboration. Cancer. (2009) 115:1224–33. doi: 10.1002/cncr.24135 19156917

[13] Milojevic B Dzamic Z Kajmakovic B Durutovic O Bumbasirevic U Sipetic Grujicic S. Prognostic impact of preoperative anemia on urothelial and extraurothelial recurrence in patients with upper tract urothelial carcinoma. Clin Genitourinary Cancer. (2015) 13:485–91. doi: 10.1016/j.clgc.2015.03.007 25920995

[14] Agha R Abdall-Razak A Crossley E Dowlut N Iosifidis C Mathew G. STROCSS 2019 Guideline: Strengthening the reporting of cohort studies in surgery. Int J Surg. (2019) 72:156–65. doi: 10.1016/j.ijsu.2019.11.002 31704426

[15] Song B Kim JK Lee H Lee S Hong SK Byun S-S. Evaluation of histological variants of upper tract urothelial carcinoma as prognostic factor after radical nephroureterectomy. World J Urol. (2024) 42:225. doi: 10.1007/s00345-024-04878-6 38592495 PMC11003889

[16] Fan B Teng Q Sun M Wang Y Wang Y Lin Z. Assessment of therapeutic benefit and option strategy on intravesical instillation for preventing bladder cancer recurrence after radical nephroureterectomy in patients with upper urinary tract urothelial carcinoma. J Oncol. (2022) 2022:1–19. doi: 10.1155/2022/1755368 PMC9170511 35677889

[17] Li X Cui M Gu X Fang D Li H Qin S. Pattern and risk factors of local recurrence after nephroureterectomy for upper tract urothelial carcinoma. World J Surg Oncol. (2020) 18:114. doi: 10.1186/s12957-020-01877-w 32473636 PMC7261378

[18] Liu J Wu P Lai S Wang J Hou H Zhang Y. Prognostic models for upper urinary tract urothelial carcinoma patients after radical nephroureterectomy based on a novel systemic immune-inflammation score with machine learning. BMC Cancer. (2023) 23:574. doi: 10.1186/s12885-023-11058-z 37349696 PMC10286456

[19] Luo Z Yan Y Jiao B Huang T Liu Y Chen H. Prognostic value of the systemic immune-inflammation index in patients with upper tract urothelial carcinoma after radical nephroureterectomy. World J Surg Oncol. (2023) 21:337. doi: 10.1186/s12957-023-03225-0 37880772 PMC10601258

[20] Wang Q Zhang T Wu J Wen J Tao D Wan T. Prognosis and risk factors of patients with upper urinary tract urothelial carcinoma and postoperative recurrence of bladder cancer in central China. BMC Urol. (2019) 19:24. doi: 10.1186/s12894-019-0457-5 30999871 PMC6471846

[21] Cowan NC. CT urography for hematuria. Nat Rev Urol. (2012) 9:218–26. doi: 10.1038/nrurol.2012.32 22410682

[22] Raman JD Shariat SF Karakiewicz PI Lotan Y Sagalowsky AI Roscigno M. Does preoperative symptom classification impact prognosis in patients with clinically localized upper-tract urothelial carcinoma managed by radical nephroureterectomy? Urol Oncol. (2011) 29:716–23. doi: 10.1016/j.urolonc.2009.11.007 20056458

[23] Qi N Zhang J Chen Y Wen R Li H. Microscopic hematuria predicts lower stage in patients with upper tract urothelial carcinoma. Cancer Manag Res. (2018) 10:4929–33. doi: 10.2147/CMAR.S180606 PMC6205531 30425581

[24] Jakus D Šolić I Borovac JA Šitum M. The influence of the initial clinical presentation of upper tract urothelial carcinoma on histopathological tumor features. Int Urol Nephrol. (2024) 56:1335–41. doi: 10.1007/s11255-023-03883-9 38015383

[25] Sun KN Wu JH Chen ZH He YJ Chen YL Hu JZ. Predictive value of flank pain and gross hematuria on long-term survival in patients with upper tract urothelial carcinoma treated by radical nephroureterectomy. Clin Med Insights Oncol. (2023) 17:11795549221147993. doi: 10.1177/11795549221147993 36685988 PMC9846590

[26] Lin T-W Lee H-Y Yang S-F Li C-C Ke H-L Li W-M. Perineural invasion is a powerful prognostic factor for upper tract urothelial carcinoma following radical nephroureterectomy. Ann Surg Oncol. (2022) 29:3306–17. doi: 10.1245/s10434-021-11265-7 34994908

[27] Li X Ma X Tang L Wang B Chen L Zhang F. Prognostic value of neutrophil-to-lymphocyte ratio in urothelial carcinoma of the upper urinary tract and bladder: a systematic review and meta-analysis. Oncotarget. (2017) 8:62681–92. doi: 10.18632/oncotarget.17467 PMC5617540 28977980

[28] Shao Y Li W Wang D Wu B. Prognostic value of preoperative lymphocyte-related systemic inflammatory biomarkers in upper tract urothelial carcinoma patients treated with radical nephroureterectomy: a systematic review and meta-analysis. World J Surg Oncol. (2020) 18:273. doi: 10.1186/s12957-020-02048-7 33097052 PMC7585317

[29] Lm C ZW. Inflammation and cancer. Nature. (2002) 420. doi: 10.1038/nature01322 PMC2803035 12490959

[30] Michelson N Rincon-Torroella J Quiñones-Hinojosa A Greenfield Jp. Exploring the role of inflammation in the Malignant transformation of low-grade gliomas. J neuroimmunology. (2016) 297. doi: 10.1016/j.jneuroim.2016.05.019 27397086

[31] Rink M Sharifi N Fritsche Hm Aziz A Miller F Kluth La. Impact of preoperative anemia on oncologic outcomes of upper tract urothelial carcinoma treated with radical nephroureterectomy. J Urol. (2014) 191. doi: 10.1016/j.juro.2013.09.010 24036235

[32] Morizane S Iwamoto H Masago T Yao A Isoyama T Sejima T. Preoperative prognostic factors after radical nephroureterectomy in patients with upper urinary tract urothelial carcinoma. Int Urol Nephrol. (2013) 45. doi: 10.1007/s11255-012-0347-1 23229166

[33] Weng P-H Hung K-Y Huang H-L Chen J-H Sung P-K Huang K-C. Cancer-specific mortality in chronic kidney disease: longitudinal follow-up of a large cohort. Clin J Am Soc Nephrol. (2011) 6:1121–8. doi: 10.2215/cjn.09011010 PMC3087779 21511834

[34] Chen J-S Lu C-L Huang L-C Shen C-H Chen SC-C. Chronic kidney disease is associated with upper tract urothelial carcinoma: A nationwide population-based cohort study in Taiwan. Medicine. (2016) 95:e3255. doi: 10.1097/md.0000000000003255 27057873 PMC4998789

[35] Hung Ph Shen Ch Chiu Yl Jong Ic Chiang Pc Lin Ct. The aggressiveness of urinary tract urothelial carcinoma increases with the severity of chronic kidney disease. BJU Int. (2009) 104. doi: 10.1111/j.1464-410X.2009.08636.x 19549259

[36] Mori K Janisch F Mostafaei H Lysenko I Kimura S Egawa S. Prognostic value of preoperative blood-based biomarkers in upper tract urothelial carcinoma treated with nephroureterectomy: A systematic review and meta-analysis. Urologic Oncol. (2020) 38. doi: 10.1016/j.urolonc.2020.01.015 32088103

