# Supplementary material for: A new nomogram for predicting extraurothelial recurrence in patients with upper urinary tract urothelial carcinoma following radical nephroureterectomy
Source: Front Oncol. 2024 Nov 6;14:1442168. doi: 10.3389/fonc.2024.1442168 (PMC11576284; doi:10.3389/fonc.2024.1442168)
Supplement: Supplementary file 1 [file Table1.docx]

Table S1 Variance inflation factor between variables.

| Variables | VIF |
| --- | --- |
| \| T \| \| --- \| | 1.242 |
| \| N \| \| --- \| | 1.110 |
| \| Hematuria \| \| --- \| | 1.025 |
| \| Grade \| \| --- \| | 1.237 |
| \| Invasion \| \| --- \| | 1.198 |
| \| NI \| \| --- \| | 1.192 |
| \| HB \| \| --- \| | 1.061 |
| \| NLR \| \| --- \| | 1.018 |

VIF: Variance inflation factor.
